# Gastric Metastasis of Ectopic Breast Cancer Mimicking Axillary Metastasis of Primary Gastric Cancer

**DOI:** 10.1155/2014/232165

**Published:** 2014-12-11

**Authors:** Selami Ilgaz Kayılıoğlu, Cihangir Akyol, Ebru Esen, Cevriye Cansız-Ersöz, Akın Fırat Kocaay, Volkan Genç, İlknur Kepenekçi, Seher Demirer

**Affiliations:** ^1^Department of General Surgery, Ankara Numune Research and Training Hospital, 06100 Ankara, Turkey; ^2^Department of General Surgery, Ankara University School of Medicine, 06100 Ankara, Turkey; ^3^Department of Surgical Oncology, Ankara University School of Medicine, 06590 Ankara, Turkey; ^4^Department of Medical Pathology, Ankara University School of Medicine, 06100 Ankara, Turkey

## Abstract

Ectopic breast tissue has the ability to undergo all the pathological changes of the normal breast, including breast cancer. Gastrointestinal metastasis of breast cancer is rarely observed and it is very difficult to differentiate gastric metastases from primary gastric cancer. We present a case of 52-year-old female, who suffered from abdominal pain. Physical examination showed a palpable mass in the left anterior axilla and computerized tomography revealed gastric wall thickening with linitis plastica. When gastroscopic biopsy showed no signs of malignancy, excisional biopsy was performed in the left axilla. Histological examination revealed invasive lobular carcinoma of the breast, consistent with ectopic breast cancer. Further gastroscopic submucosal biopsies and immunohistochemical studies revealed gastric metastases of invasive lobular carcinoma. Axillary ectopic breast tissue carcinomas can mimic axillary lymphadenopathies. Additionally, gastric metastasis of breast cancer is an uncommon but possible condition. To the best of our knowledge, this is the first report of ectopic breast cancer with gastric metastasis.

## 1. Introduction

Ectopic breast tissue may be observed in any site along the milk line, from the axilla to the inguinal region [1, 2]. Although the incidence of ectopic breast tissue is not clearly known, it is believed to be 1% in the general population [1]. Ectopic breast tissue has the ability to undergo all the pathological changes of the normal breast, including breast cancer. Carcinoma occurs more frequently in ectopic breast tissue occurring in the axilla than in extra-axillary ectopic breast tissue. Local and distant metastases of breast cancer to the lymph node, bone, pulmonary, liver, and brain are frequently seen. Gastrointestinal metastases are rarely observed [3] and when a malignant lesion is detected in the stomach, it is very difficult to differentiate gastric metastasis of breast cancer from primary gastric cancer clinically, endoscopically, radiologically, and pathologically. Some of these patients undergo gastrectomy, with a preoperative diagnosis of primary gastric cancer. As gastric metastasis of breast cancer is rarely encountered, literature review failed to demonstrate gastric metastases of cancer originating from ectopic breast tissue.

## 2. Case Presentation

**Figure 1:**
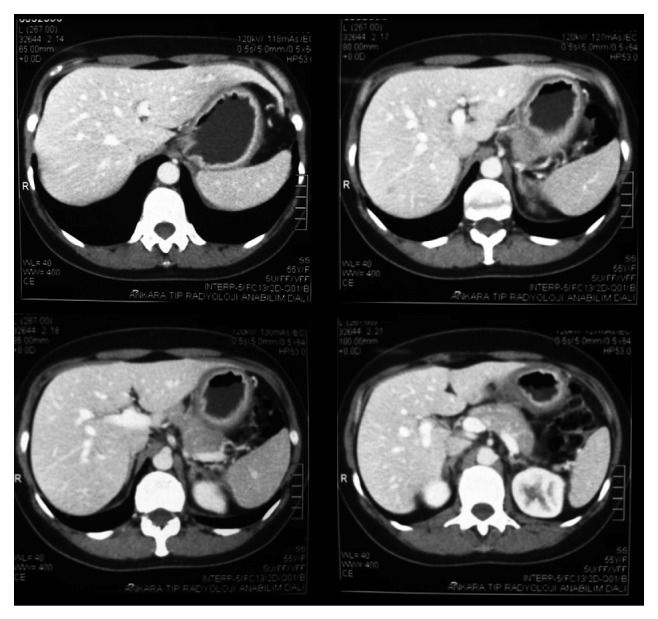
Computerized tomography image of 52-year-old patient. Gastric wall thickening of linitis plastica can be seen.

**Figure 2:**
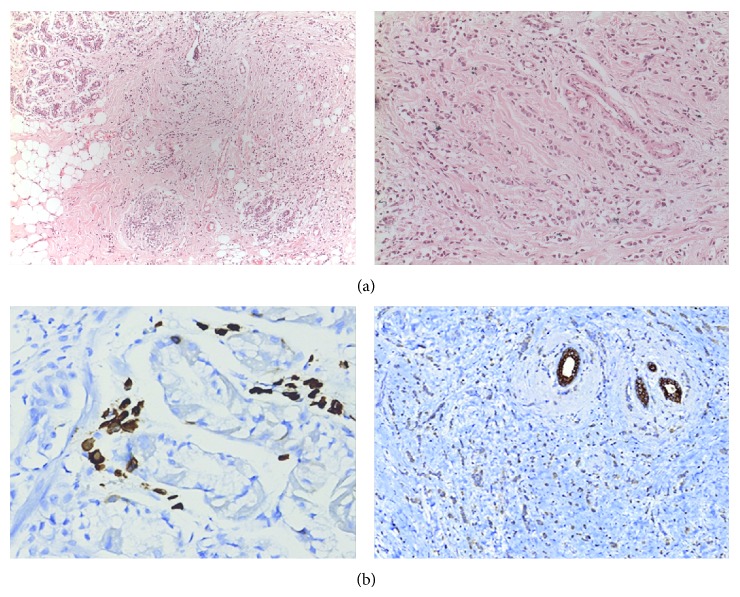
Histological images of ectopic breast tissue, gastric mucosa. (a) Left: tumor infiltration in ectopic breast tissue. H&E ×10. (a) Right: small noncohesive tumor cells individually dispersed or arranged in a single-file linear pattern in ectopic breast tissue. H&E ×20. (b) Left: GCDFP15 is focally positive in neoplastic cells. GCDFP15 ×40. (b) Right: strong positivity seen in regular duct epithelium. Loss of staining in neoplastic cells. E-cadherin ×20 (H&E: Hematoxylin and Eosin).

**Figure 3:**
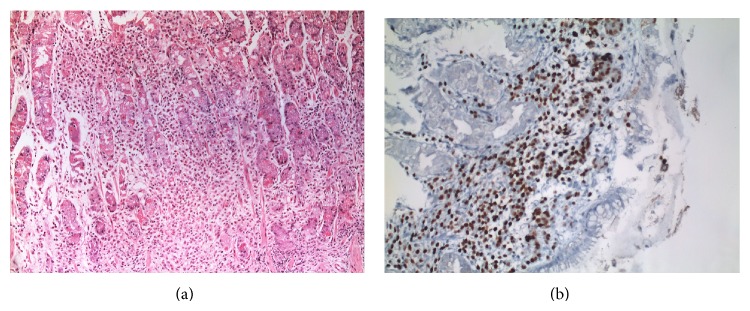
(a) Gastric mucosa infiltrated by cords of metastatic lobular carcinoma cells. H&E ×10. (b) Estrogen receptor positive tumor cells in gastric lamina propria. H&E ×20 (H&E: Hematoxylin and Eosin).

A 52-year-old female patient presented with abdominal pain and weight loss for 6 months. On physical examination, mobile mass measuring 3 × 2.5 × 1 cm with smooth margins was palpated in the left anterior axillary line. Gastric wall thickening with linitis plastica appearance was detected on computerized tomography (Figure 1). Upper gastrointestinal system endoscopy showed atrophic mucosal changes in the stomach, and histological examination did not show any malignant cells. With a preoperative diagnosis of axillary lymph node metastasis from gastric cancer, the patient underwent excisional biopsy of the axillary mass. Histological examination showed invasive lobular carcinoma arising in ectopic breast tissue. The tumor was composed of noncohesive cells individually dispersed or infiltrating in a single-file linear pattern resembling “Indian files,” within a fibrous stroma (Figure 2(a)). Immunohistochemical staining revealed the tumor cells to be diffusely strong positive for progesterone receptor (PR) and faintly positive for estrogen receptor (ER). As the morphological appearance strongly indicated lobular carcinoma, E-cadherin and GCDFP-15 were studied. Strong E-cadherin positivity was seen in regular duct epithelium, and on the other hand, loss of staining was observed in neoplastic cells (Figure 2(b) (left)). Moreover GCDFP15 was focally positive in neoplastic cells (Figure 2(b) (right)). Endoscopy was repeated and submucosal biopsy samples were obtained. Histological examination showed atypical cells containing small, round, oval-shaped nucleoli with faintly eosinophilic or clear cytoplasm (Figure 3(a)). Immunohistochemical staining showed the tumor cells to be ER and PR positive (Figure 3(b)), cytokeratin (CK) 20 negative, and CK 7 intensely positive, which is consistent with metastatic lobular carcinoma. Bilateral mammography imaging was performed, demonstrating nodular dysplastic changes in fibroglandular tissue, nodular opacities superimposed with fibroglandular tissue and in the left breast upper-outer quadrant, and conglomerated microcalcifications with low density. On breast ultrasonography, the upper-outer quadrants of both breasts were more intense. Hypoechoic multicentric solid lesions measuring 11 × 10 mm and 7 × 4 mm in the left breast and 12 × 11 mm in the right breast were present. In the left axillary region, multiple lymph nodes with thick cortices measuring 15 × 10 mm were seen. Ultrasonography guided large-core needle biopsy was performed for the lesions in the left breast. Histological examination showed no signs of malignancy. The patient was sent to the Medical Oncology Department in order to plan further treatment. The patient is followed up for 12 months. No signs of malignancy in the left breast were observed in this period.

## 3. Discussion

Breast development begins in the 4th embryologic week. Milk lines originating from the ectoderm appear on the anterior abdominal wall in the 5th-6th embryological weeks and gradually regress, except in the pectoral area. When problems arise during the process of regression of the mammary fold in the embryological period, impairments in the number and location of the breasts are observed. While the presence of supernumerary nipples alone is called polythelia, the presence of supernumerary breast is called polymastia. Ectopic breast tissue that does not contain the nipple or areola, but only glandular breast tissue, is called aberrant breast tissue. Ectopic breast tissue cancers account for 0.3–0.6% of all breast cancers and generally originate from the axilla [4]. Otherwise, it may also be seen in the sternal area, subclavian region, infraclavicular region, xiphosternal junction, epigastrium, and labia majora. Ectopic breast cancer generally involves women aged above 40 years. While the male/female ratio is 0.01 for primary breast cancer, it is increased to 0.035 in ectopic breast cancer. All other risk factors seem to be similar to those of primary breast cancer [5]. Histologically, most adenocarcinomas arising in aberrant breast tissue have been of the ductal type. Other subtypes also have been described, including medullary, papillary, and lobular carcinomas. In ectopic breast cancer arising in the axilla, metastasis to the supraclavicular lymph nodes that ensures the lymphatic drainage of this region should be considered regional lymph node metastasis rather than distant metastasis. Contrarily, while internal mammary lymph node metastases are considered nodal metastases in primary breast cancer, they should be regarded as distant metastases in ectopic breast cancer arising in the axilla. Treatment consisting of locoregional surgery (local excision and axillary dissection) and radiotherapy is considered enough. Endocrine therapy and/or chemotherapy should be added [5].

The gastrointestinal tract is a rare site of metastasis. In a postmortem series of 1010 patients, 17 subjects had gastric metastases (1.7%) and were most commonly from primary tumors such as breast cancer, lung cancer, and melanoma [6]. As seen in the case presented, gastric metastasis of breast cancer is manifested by nonspecific symptoms, such as dyspepsia, anorexia, and epigastric pain. Endoscopically and radiologically, it frequently mimics linitis plastica [7]. The typical finding is diffuse intramural infiltration of the gastric wall by the tumor cells. The most commonly observed metastatic breast cancer is invasive lobular carcinoma [7, 8]. As seen in our case, endoscopic findings may not be helpful in the diagnosis. Although the lamina propria was later shown to be infiltrated, no signs of tumor were spotted in gastroscopy. Because the metastatic tumor is usually limited to the submucosa and seromuscular layer, 50% of the cases have normal endoscopic findings [9]. Unfortunately, due to the presence of signet ring cells, lobular carcinoma of the breast is commonly confused with diffuse adenocarcinoma of the stomach. Therefore, an immunohistochemical analysis is required for the differential diagnosis. Several studies have shown almost uniform negativity for ER in primary gastric carcinomas is commonly detected. In contrast, one study demonstrated that up to 28% of these tumors may be ER positive, with a focal weak-to-moderate staining intensity [10]. However, in our case, ER staining was strong and diffuse. ER alpha, which is a second generation antibody, may be helpful in the differential diagnosis [11]. ER negativity in the primary tumor may create serious challenges in the diagnosis. In this case, GCDFP-15 (gross cystic disease fluid protein-15) may be a significant marker, useful in the diagnosis of metastatic breast cancer [12]. CK 20 is positive in gastric, colorectal, pancreatic, and transitional cell carcinomas, while it is not usually observed in any breast carcinomas [13]. In contrast, CK 7 is positive in 90% of breast carcinomas and also in 50–64% of primary gastric adenocarcinomas [14, 15].

Consequently, CK 7 and CK 20 expression patterns are very useful in diagnosing metastatic carcinomas of uncertain origin. While approximately 30% of gastric adenocarcinomas are CK7+/CK20+, 20% are CK7−/CK20+, 10% are CK7−/CK20−, and 20% are CK7+/CK20− [15]. Therefore, a panel of markers including ER, GCDFP-15, and CK 7/CK 20 may be used in the differential diagnosis of breast and gastric carcinoma in difficult cases. Gastric metastasis of breast cancer is considered to be a systemic disease. Anticancer chemotherapy and endocrine therapy are effective, but surgery has no effect on the prognosis. In the series by McLemore et al. [16], the mean survival time was 28 months. In our case, the follow-up period is about 24 months.

In conclusion, all kinds of diseases observed in primary breast tissue may also develop in ectopic breast tissue. Metastasis of ectopic breast cancer to the stomach, which is a rarely encountered metastatic site, is occasionally confused with primary gastric cancer. In our case, which is the first case of this kind in the literature, the initial presentation suggested the diagnosis of gastric cancer, but further examination revealed the accurate diagnosis of metastatic ectopic breast cancer.
